# Assessment of Data Fusion Algorithms for Earth Observation Change Detection Processes

**DOI:** 10.3390/s16101621

**Published:** 2016-09-30

**Authors:** Iñigo Molina, Estibaliz Martinez, Carmen Morillo, Jesus Velasco, Alvaro Jara

**Affiliations:** 1 ETSITGC, Technical University of Madrid, 28031 Madrid, Spain; cmorillo@topografia.upm.es (C.M.); jesus.velasco@upm.es (J.V.); 2 ETSIInf, Technical University of Madrid, 28031 Madrid, Spain; mariaestibaliz.martinez@upm.es (E.M.); amjgalan@gmail.com (A.J.)

**Keywords:** change detection, radiometric normalization, thresholding, informational metrics, sensor fusion, Support Vector Machine, quality assessment

## Abstract

In this work a parametric multi-sensor Bayesian data fusion approach and a Support Vector Machine (SVM) are used for a Change Detection problem. For this purpose two sets of SPOT5-PAN images have been used, which are in turn used for Change Detection Indices (CDIs) calculation. For minimizing radiometric differences, a methodology based on zonal “invariant features” is suggested. The choice of one or the other CDI for a change detection process is a subjective task as each CDI is probably more or less sensitive to certain types of changes. Likewise, this idea might be employed to create and improve a “change map”, which can be accomplished by means of the CDI’s informational content. For this purpose, information metrics such as the Shannon Entropy and “Specific Information” have been used to weight the changes and no-changes categories contained in a certain CDI and thus introduced in the Bayesian information fusion algorithm. Furthermore, the parameters of the probability density functions (pdf’s) that best fit the involved categories have also been estimated. Conversely, these considerations are not necessary for mapping procedures based on the discriminant functions of a SVM. This work has confirmed the capabilities of probabilistic information fusion procedure under these circumstances.

## 1. Introduction

Change Detection (CD) is a process by which the differences in the state of an object or phenomenon are identified upon observation at different times; in other words, the quantification of temporary effects using multi-temporal information [1]. Determining changed areas in images of the same scene taken at different points in time is of considerable interest given that it offers a large number of applications in various disciplines [2], including: video-surveillance [3], medical diagnoses and treatment [4], vehicle driving support [5] and remote detection.

Specifically, Change Detection in images recorded by means of remote sensors is considered a branch of technology called remote sensing. What contributes to this fact is the temporal resolution of satellite sensors, the inherent orbit repetitiveness of which means images of a certain target area can be recorded with a certain regularity. Moreover, the content improvement of spatial technology has led to the development of high-resolution sensors, which supply large volumes of data containing high quality information. Many CD techniques have been developed in the area of remote sensing, which have been compiled in excellent works [1,2,6,7,8,9]. Although there is a large variety of change detection algorithms in the literature applied to different types of images, none stand out as being able to satisfy all possible problems.

The Change Detection process is extensively described in the work [2]. Generally, at a first stage a Change Detection Index (CDI) is generated, and then by means of thresholding, a Change Detection Map (or binary image) is derived. With respect to this thresholding process, it can be accomplished automatically, as it has already been performed in previous studies [10] based in the algorithms described by [11].These strategies have also been applied in part of this research.

The analysis of the temporal changes in a given geographic area may be based on an analysis of two scenes of the area taken on two different dates (bitemporal) or on a multi-temporal series. In any case, it is a topic of great interest in image processing and interpretation [12] and the main aim lies in the capacity to discriminate real significant changes or true positives, which are required for a specific application.

Basically, CD techniques are grouped according to two approaches: supervised and unsupervised [13]. The first group includes methods that require reference land cover information, which is necessary to obtain thematic maps exhibiting the transitions between the types of land cover changes; they are based on the use of supervised classifiers [14]. The disadvantages of these methods include: the human efforts and time involved in obtaining the reference information and the possible errors committed when processing the classification. On the other approach, unsupervised methods do not need reference data to generate a binary map of the change/no-change areas, and a priori they seem to be more attractive from an operational perspective especially for the analysis of large data sets [8]. A compromise between both methods can be achieved, where some training operations might be automated. In these situations, they may be referred to as hybrid training methods, which will be the case for one of the suggested procedures of this work. Moreover, one fundamental problem is obvious the fact that significant changes targeted by the analysis and final application are inevitably influenced by others, which are not so significant [15], and which can largely influence the precision of the results obtained. For this reason, it is important to pre-process the images [16,17,18,19], because differences may exist in the record of the multi-temporal image (image radiometry and/or geometry), which produce false positives in the CD results [20]. To this end, the use of relative or absolute radiometric normalization techniques is also debated. The latter leads to the reduction of atmospheric effects that affect the images in the data set, among other issues [21]. There is no one ideal approach applicable in all cases.

From a change detection perspective, using images with good spatial quality in which the changing areas can be adequately defined is a major advantage. However, higher spatial resolution in the images sometimes involves processing a large quantity of information, which are considered computational heavy images, when the aim is to study relatively extensive geographic areas without using process parallelization or scene division strategies. Thus, a compromise must be reached between the effectiveness of the method used and the computational cost. For this reason, panchromatic images are considered to be a suitable alternative.

In spite of the fact that there are a large number of CD techniques and methods as a result of all of the work and studies already completed, it continues to be an active research topic. The question posed in this paper is related to the optimization of these processes based on the ideas outlined below. In order to compensate for the insufficiency of a single source of remote information during CD and combine the different complementary properties of different sensors, Zeng et al. [22] suggest applying fusion algorithms to improve the results in these processes. Other proposals also exist in this area such as the one offered by [23], who explore the advantages of combining different traditional CD methods in order to obtain more accurate and reliable results based on individual methods. Some new research lines have been aimed at developing multi-feature fusion procedures [24] for visual recognition in multimedia applications. This research seeks to combine multi-source sets or different remote sensing sensors [20,22,25,26] to extract the best entities corresponding to change or no-change areas from them. On the other hand, other authors [10,27,28], suggest using the informational complementarity of the different change detection indices (CDI) obtained from a single multi-temporal pair of data. The fusion procedures may be of different types. Le Hegarat et al. [27] propose methods based on the Dempster-Shaffer Theory, [10] apply statistical or probabilistic analysis models based on the multi-sensory Probabilistic Information Fusion theory, whereas [28] use fusion techniques based on Discrete Wavelet Transform (DWT). Other possibilities could be Neuronal Networks [29,30], and fuzzy logic [31]. A complete list of these methods can be found in [32].

Based on the previous accomplished related work, this study is motivated by the fact that a single source or CDI does not reflect all the changes occurred on a particular landcover, thus the need to explore fusion algorithms in order to deal with the complementary information of different sources or CDIs in such a process, and hence overcome this insufficiency. In this case the contribution of each source must also be evaluated in order to potentiate its best change/no-change informational content, which during the CD process might also help to optimize the final result or change map. Moreover, when using parametric models, each CDI change/no-change categories must be properly parameterized according to a best fitting probabilistic function. Then, as a consequence of these three main motivations, this paper focuses on the analysis of probabilistic information fusion applied to CD, as they are considered sufficiently suitable for taking into account these different problems. Two model types were chosen: a model based on the sum of probabilities a posteriori, as seen in [10], and another based on the logarithm of their products. CDIs comprise the multi-source or multi-sensor information that is fused using these two models. For this reason, one important issue to be taken into account when considering different information sources in fusion processes is to verify the contribution of each source (weight) in said process. This contribution can be assigned ad-hoc or other analytical means can be used to weigh each CDI or the categories they contain based on the informational content. The weight assignment issue has also been considered in different papers but with different approaches. For example, [33] applied the Multivariate Alteration Detection (MAD) method for a CD problem with hyperspectral images where the weights were re-assigned based on the procedural iterations meaning the assignments are inherent to the MAD method. This algorithm has also been applied by [34] as part of a multi-source change detection scenario similar to the one described in [23]; however, the CDIs that intervene in other multi-source scenario detection methods are not affected by any type of weighting. Unlike the MAD method, one fundamental objective of this study was to evaluate a set of informational metrics based on Shannon entropy susceptible to supplying the adequate weights to duly weight the change/no-change categories of the CDIs considered in the corresponding probabilistic models. Another important part of this work and of probabilistic information fusion procedures has to do with the probability functions and the corresponding parametrization of the CDI categories that intervene in this process; hence the need to evaluate the functions and parameters that best suit these categories. The results provided by this method are contrasted with nonparametric algorithms based on Support Vector Machines (SVM). In this work, these different algorithms are also regarded to as fusion methods since they generate a unique CD Map (output) from different CDIs (inputs), such as in the probabilistic methods. Finally, another important pillar of this work consisted of comparing performance as concerns the flexibility and reliability of the probabilistic procedure versus the SVM-based one. In summary, the novelty, merits and key contributions of this work are manifold.

False alarms in a CD process can be efficiently reduced by applying a robust image normalization process, aimed at better identification of no-change zones. In this work a new methodology is applied, which combines a relative correction with an absolute image radiometry transfer based on zonal features extracted automatically.

For change map generation, two fusion procedures, parametric and nonparametric, for remote sensing image Change Detection are applied and assessed.

For the parametric procedure case, the evaluation of the contribution of the two categories of each CDI is required. Three information metrics are suggested and contrasted. An important fact about these metrics is that they can be determined analytically, which can be considered as a novel contribution in information fusion applied to image change detection.

For each CDI categories, the best fitting probability function and statistical parameters must also be supplied, this avoids using a generic probability function, e.g., Gaussian model, which might be incorrect in most cases, and is also aimed at improving the results of the fusion process. This also conforms a novelty in this particular field of information fusion.

Traditional accuracy estimations methods and metrics are not totally suitable for quality assessment in high resolution segmented datasets, as it is the case in change maps derived from high resolution sensor images, therefore two different object based metrics are proposed in this work.

The rest of this paper is organized as follows: in Section 2, first a description of the image datasets involved in the CD processes is given. Then, the related work with the corresponding proposed CD framework is suggested, which includes two specific, parametric and nonparametric, information fusion algorithms. Finally, in this section, a different approach for assessing the CD results is also proposed. In Section 3, the experiments and results of the suggested methodology are presented, and Section 4 deals with the discussion of these results. Finally, the conclusion is drawn in Section 5.

## 2. Materials and Methods

**Figure 1:**
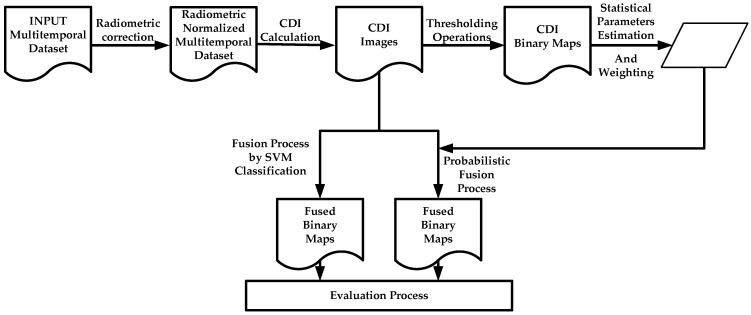
Global change detection suggested methodology.

This section describes all the data used to carry out the proposed CD processes. The generation of change maps by means of the suggested information fusion methodologies is based on two basic phases. The first needs to pre-process the image dataset, so that they are adapted by transferring the radiometric conditions from one image to another. This particular question is addressed in Section 2.2. The second phase is aimed at obtaining change maps through information fusion processes. Two options arise. First a probabilistic information fusion methodology is optimized with the estimation of statistical parameters of different probability functions (Section 2.5), which also includes an analytical procedure for weighting of the change/no-change categories of each CDI (Section 2.4). The second option, also included in Section 2.5, evaluates a supervised SVM classification for generating a single change map form different CDIs. In this work, the fusion processes required for obtaining a single change map, are considered late fusions, as they fuse the generated information at a late stage of the process, which is differs from early fusion, where the fusion is made during data pre-processing. Finally, an alternative to traditional methods for accuracy estimation or quality assessment of the change maps is suggested in Section 2.6. The experiments have been conducted on image datasets described in Section 2.1. The overview of the proposed CD framework is illustrated in Figure 1.

### 2.1. Study Area and Data

Two geographic areas have been selected for this study. They are located in central and eastern Spain, respectively. The major land-cover/land-uses of the two areas are urban and rural categories. The changes are produced from the conversion of rural into residential and/or commercial land-uses, although other changes are also observed due to seasonal changes in crops. The most remarkable changes are the construction of new infrastructures.

**Figure 2:**
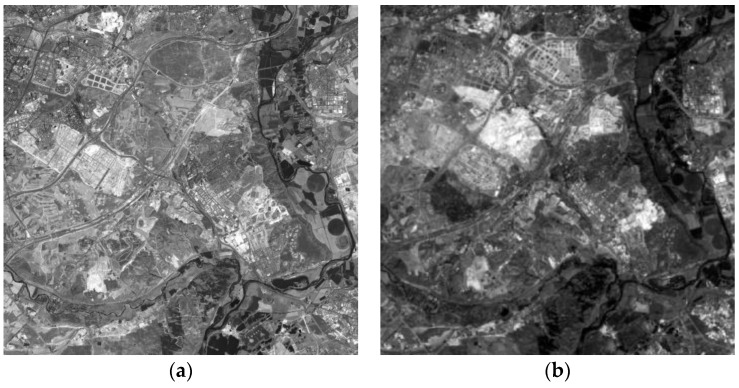
SPOT5-PAN (© SPOT image) multitemporal DS1: (**a**) Acquisition date 24 July 2005; (**b**) Acquisition date 17 July 2008.

The research area 1, referred to as dataset 1 or DS1, is located in the southern part of the city of Madrid (Region of Madrid). The data used for the CD analysis are two panchromatic images (5846 × 5760 pixels or 210.5 km^2^) registered by the SPOT5 HRG Sensor, and acquired the 24 July 2005 and 17 July 2008 (Figure 2), respectively.

Their reference in the SPOT image grid system is 269/35 (K/J), and the UTM coordinates of the center of this area are 452,914 E, 4,474,699 N (zone 30, WGS84). The spatial resolution is 2.5 m, the spectral interval is 0.70–0.90 µm, and the radiometric resolution is 8 bits.

**Figure 3:**
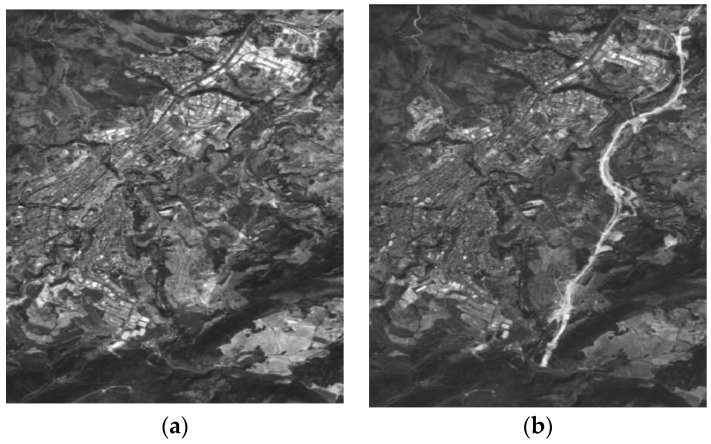
SPOT5-PAN (© SPOT image) multitemporal DS2: (**a**) Acquisition date 14 August 2005; (**b**) Acquisition date 10 August 2008.

The research area 2, referred to as data set 2 or DS2, is located near to the metropolitan region of Alicante (Region of Valencia). This data set is also made up two panchromatic images (1781 × 2637 pixels or 29.2 km^2^) of the same sensor system. Their reference in the SPOT image grid system is 272/41 (K/J). The images of this second dataset have been acquired on 14 August 2005 and 10 August 2008 (Figure 3), respectively. The UTM coordinates of the center of this area are 418,233 E, 4,289,645 N (zone 30, WGS84).

### 2.2. Radiomeric Normalization

Different independent factors caused by land covers may significantly affect the reflectance measured by a sensor. They include the sensor calibration the solar elevation, the atmospheric conditions and the topography. This can be treated as a domain adaptation problem as suggested in [35,36]. For images registered by remote sensing sensors, it is necessary to adapt the radiometric conditions from one image to another in order to compensate for these effects on the temporal series of images and thus avoiding dissimilarities in non-changed landcovers. For this purpose, radiometric normalization techniques reduce the radiometric variation induced on the lands covers in order to improve the territory CD processes [37].

In general, there are two main groups of procedures used for radiometric normalization of remote sensing images. They are divided into relative correction [38,39] and absolute correction [21] methods. As concerns the former, these methods cannot be considered correction methods as they do not take into account atmospheric conditions or the solar irradiance when the image is taken but rather attempt to mitigate or minimize the effects with respect to a reference image selected by an analyst [40]. Yuan and Eldvige [38] identified an important set of these types of methods.

On the one hand, absolute radiometric correction takes into account the atmospheric conditions that contribute to radiative transfer as well as the sensor gains and movements, solar irradiance, etc., which make it possible to determine the exoatmospheric reflectance values as if they were determined on the land surface. Over the last few decades, a large variety of methods has been developed aimed at correcting the effects on satellite images caused by the atmosphere. They range from simple procedures such as Dark Object Substraction (DOS) [41] and Cosine Transmission correction (COST) [42] to more complex methods like 6S [43].

The choice in the field of CD has generally been to apply radiometric normalization related methods. Nonetheless, other studies [21] suggest the appropriateness of applying absolute methods such as those indicated above. The issue here is determining the degree of complexness of the procedure required for a CD case. To this end [30] indicate that contrary to what would be expected, the most sophisticated atmospheric correction methods do not provide better results in a CD process. For these situations, they recommend methods that basically reduce the effect of Rayleigh scattering. Therefore, methods such as the one indicated in [30] seem to be suitable for this purpose. A hybrid alternative combining the application of relative and absolute methods was chosen for this work. Moreover, it is proposed a novel use of automatic thresholding techniques that help better define the no-change areas, which are our Pseudo Invariant Features (PIF) or Invariant Targets [21,44,45,46].

The hybrid radiometric image normalization procedure begins with a relative method [16,45,47]. To do so, you only need the standard means and deviations of the two images comprising the dataset and decide which to use as the reference (master) based on the one that shows the broadest dynamic range [21]. The result is an adjusted image with respect to the first order statistical parameters of the master image. A CDI is calculated based on this with the algebraic operation of the difference between the pixels of the two images. The CDI calculation method is covered below in Section 2.3.

**Figure 4:**
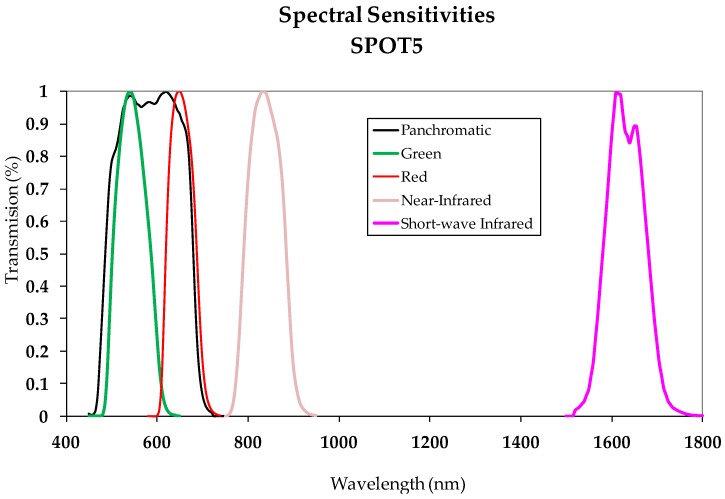
SPOT 5—spectral sensitivities (from Renza et al., 2011).

One way to automatically obtain the change/no-change binary mask is to apply an automatic thresholding process to the CDI. The choice of thresholding method is decided based on the size of the CDI value range. In other words, the Kapur or Otsu procedures are suggested for images with value ranges defined by a minimum and maximum close together [11]; however, applying methods based on entropy such as Li and Shanbhag is recommended for broader value ranges [11]. The no-change zones thus obtained define the PIF or Zonal Invariant Features (ZIF). These invariant zones are the ones later used for the absolute calibration process. The two images comprising the Dataset are atmospherically corrected in a second phase. This correlation was done for the images in this study by applying what is known as the DOS method. It is believed that this method is sufficient for images acquired from panchromatic sensors with the spectral width indicated in Section 2.1, as verified in the SPOT5 HRG sensor spectral sensitivity shown in Figure 4. These same considerations are outlined in the work by [21] for the same type of data.

**Figure 5:**
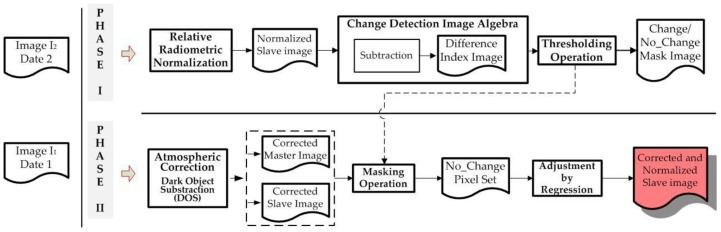
Suggested Radiometric Normalization Methodology.

Once this atmospheric correction is done, the absolute radiometric normalization between the two images of a multitemporal data set is done. To do so, the Pseudo Invariant Features, are applied, which are defined in this work as zones in the two images where no changes have occurred, so Zonal Invariant Features (ZIF). Yuan and Elvidge [45] define the “no-change pixel set” using this method. This helps to mask the probable “change pixels” in the two images. The slave image is adjusted to the master image by means of a controlled linear regression process [21,45,46,47]. The “slope” and “intercept” parameters obtained from the adjustment are used to transform the complete and atmospherically correct slave image. The “slope” and “intercept” are calculated using the covariance and standard deviations by means of the zonal invariant features, which make it possible to construct the linear transformation function that adjusts the slave image to the reference one. The different phases and operations applied with this method are summarized in Figure 5.

Finally, it is important to establish that both normalization processes (relative and absolute) are evaluated in the first and second phase (Figure 5) by applying the RMSE (Root Mean Square Error) method described in [16,45], which is expressed as Equation (1):

(1) $$\mathrm{RMSE}=\sqrt{\frac{1}{n}\sum {\left(\rho_{\mathrm{ref}}-\rho_{\mathrm{adj}}\right)}^{2}},$$

where ρ_ref_ and ρ_adj_ are, in this case, the reflectances or, as applicable, the digital values (DN) of the reference and normalized images, respectively.

### 2.3. Computation of Change Detection Indices (CDIs)

There is a great number of CDIs and each one of them can be applied to different types of images from Panchromatic vs. Multi-spectral sensors [1,2,6,7,27]. In the case of PAN images, the traditional algebra-based image indices, such as Difference CDIs and Log_Ratio, have proven to be effective meaning their use is considered in this work. The Difference CDI is calculated with the absolute difference of the pixels of the normalized bitemporal DS images (I_i_, I_j_). For the Log_Ratio CDI, the method set forth by [48] was used. It consists of calculating the log ratio between the two equally normalized images, defined by the following Equation (2):

(2) $${\mathrm{CDI}}_{\text{log rat}}=\left|\log\left(\frac{I_{i}}{I_{j}}\right)\right|=\left|\log I_{i}-\log I_{j}\right|,$$

Besides these two indices, a third index was taken into consideration for this paper based on entropic information such as Kullback-Leibler Divergence. In this case, the initial premise to be assumed is that a pixel has changed if the statistical distribution changes from one date to another. Used as the means to quantify this change, this scalar index, which maps the two estimated statistical distributions for each image (bitemporal) and the symmetric version may be known as Kullback-Leibler Distance (Kb_Leibler) [49] as expressed by the following Equation (3):

(3) $$D\left(I_{i},I_{j}\right)=D\left(I_{j},I_{i}\right)=K\left(I_{j}/I_{i}\right)+K\left(I_{i}/I_{j}\right),$$

where K designates the Kullback_Leibler divergence. This index can be calculated as the entropy between the two probability density functions of the two images. They must be known in order to compare these functions and, therefore, the image must be explored via small windows using local statistical models. The detector applied in each window of dimension selected for a Gaussian probability density with local mean parameters, μ and variance σ^2^, is calculated across the image with the following local operation Equation (4):

(4) $$\text{detector}=\frac{\sigma_{i}^{4}+\sigma_{j}^{4}+{\left(\mu_{i}-\mu_{j}\right)}^{2}{\left(\sigma_{i}^{2}+\sigma_{j}^{2}\right)}^{2}}{2\sigma_{i}^{2}\sigma_{j}^{2}}-1,$$

A substantial part of this research is applying information fusion procedures for CD. Based on the idea already expressed where each CDI contains complementary information, one of the essential tasks is to explore each of their contributions, by means of reliability factors or weights, to ideally integrating this information in an information fusion process as if it were a sensory management system. Notwithstanding, it must be mentioned that this proposal is only applied to the Bayesian information fusion procedure which will be described further below in Section 2.5, as said weighting is not applicable a priori in the processes based on SVM.

The next step consists of obtaining the binary change maps. The most suitable thresholding method is chosen for each CDI. This is done in the same way as indicated for the radiometric normalization process. In doing so, the criteria outlined by [10,11] was taken into consideration whereby certain algorithms are more appropriate for some ranges and image value distributions. The idea behind this binarization process is to supply a base to obtain essential information for the proper execution of the probabilistic information fusion models. Thus, each binary image obtained previously makes it possible to mask the change/no-change areas in each CDI and after that, determine the contribution of each CDI and/or its categories by calculating the informational metrics on the one hand and extract the parameters of the distribution functions that define each one of these categories in each CDI on the other hand. This latter is in fact the training process required for characterizing change and no-change classes, and can be understood as a hybrid training procedure, contrary to SVM methodology, which the training must be manually driven.

### 2.4. Informational Content Analysis

This paper focuses on different theoretical approaches where different theoretical informational metrics are compared to prioritize the inclusion of the information contained in each CDI. When measurements are selected to be included in a Bayesian inference process, the ones that provide the most information on the status of the object observed (in this case, the change/no-change categories) must be considered so the distributions a posteriori contain or reproduce most of the information. One broadly accepted measurement of such information is the statistical entropy introduced by Shannon [50], the general form of which is Equation (5):

(5) H(X)=−∫p(x)log[p(x)]dx,

At the same time, when an observation x of a random variable X is conditioned by the existence of another measurement y_i_, another way to write the entropy is by means of what is known as conditional entropy (or mutual information, [51]), as expressed by Equation (6):

(6) $$H\left(X/Y=y_{i}\right)=-\int p\left(x/y_{i}\right)\log\left[p\left(x/y_{i}\right)\right]dx,$$

The increased information due to the existence of y [52], which equals the change in the uncertainty that occurs in x when y_i_ is observed is expressed by the difference Equation (7):

(7) $$\Delta I=H\left(X\right)-H\left(X/y_{i}\right)=-\int p\left(x\right)\log\left[p\left(x\right)\right]dx-\int p\left(x/y_{i}\right)\log\left[p\left(x/y_{i}\right)\right]dx,$$

This expression introduced by [53] is also known as specific information and is designed in this work by I_2_, and an increase in this value means a category can be easily predicted given the observation y_i_ or the increased information due to said observation.

The three informational metrics mentioned in this section were taken into account to establish a weighting system based on the information content of the CDIs. Firstly, weights are established for each CDI based on the entropic information for each one of them. Then, other weights are calculated for each one of the change/no-change categories based on the conditional entropy and maximizing of the mutual information as suggested by [54]. These weights are calculated by applying the “self-ranking” concept described in [55]. Once these weights are determined, they can be introduced and/or used for probabilistic information fusion models. The aim is for each weight to explicitly reflect the contribution expressed by each of the metrics used, and satisfying the condition that the sum of the weights for the corresponding metrics is always equal 1. This particular question has already been addressed in [56] for CD problems. However, the novel idea with respect to the bibliography consulted is that this work seeks an analytical solution for the efficient determination of values that duly scale each CDI based on the information each one of them contributes.

### 2.5. Change Detection Procedures Using Information Fusion Strategies

The idea that supports the following proposal is based on the currently ever-greater increase in the high number of operational sensors and the fact that the information on a scene can be captured using different types of sensors in such way that one mean to take advantage of these different sources of information is by a fusion procedure. Of the various procedures [57], the probabilistic information fusion algorithm was chosen when doing this work. It is held up by the Bayesian estimation theory and requires knowledge of the functions of density/distribution to express the uncertainty of the data. In the general case of multisensor fusion [58], considering a set of images X^1^, …, X^p^, recorded by one or several sensors and the types of scenes designated by C_i_, the probabilistic fusion consists of assigning each pixel to the category that maximizes the probabilities a posteriori Equation (8):

(8) $$P(C_{i}/X^{1},\ldots ,X^{p})=\frac{P(X^{1},\ldots ,X^{p}/C)P(C_{i})}{P(X^{1},\ldots ,X^{p})},$$

where P(C_i_) is the a priori probability. This approach is similar to the one described by [59] known as the consensus theory. It adds that the most commonly used rule of consensus for multiple sources is the “Linear Opinion Pool” (LOP) [60], which converts the Equation (8) in the membership function by means of the Equation (9):

(9) $$F(x)=\sum_{i=1}^{n}\lambda_{i}P(C_{i}/X^{p})\;\mathrm{with}\;\sum_{i=1}^{n}\lambda_{i}=1,$$

where (λ_i_) are the specific weights of the sources that control the influence thereof and are associated with them to quantitatively express their reliability. Another rule of consensus is “Logarithmic Opinion Pool” (LOGP), the membership function of which is written as Equation (10) in this case:

(10) $$L(x)=\prod_{i=1}^{n}P(C_{i}/X^{p}{)}^{\lambda_{i}},$$

which can be better written [58] as Equation (11):

(11) $$\log(L(X))=\sum_{i=1}^{n}\lambda_{i}\log(P(C_{i}/X^{p})),$$

where (λ_i_) also reflect the reliability of the same sources. Having previously mentioned that different CDIs contribute complementary information [10,28] suggest applying Equations (9) and (11) to different CDIs deriving from a multitemporal set of images acquired by one or several sensors in order to enhance the result of a change map. This study shall work with a single type of sensor, but with different CDIs derived from this latter.

Another important issue refers to the analysis of the distribution function that best fits the change/no-change categories of a certain CDI. In general, behaviors have always been considered with Gaussian parameters when applying these types of models. Nonetheless and even when considering this fact, it is important to verify and confirm this assumption given that it is very likely depending on the different cases that the values of said categories better fit with other probability functions. If this were true, the results would be expected to also be affected. Therefore, this paper has taken another two functions of density/distribution into account, which are exponential, and Weibull. The exponential one is modeled by the parameter (λ) whereas the Weibull one consists of two parameters α and β which scale and model the distribution of the values of a single variable, respectively. The selection of an exponential function is due to the fact that the distribution of the values of change/no-change categories generally show exponentially opposite behavior. On the other hand, the selection of the Weibull function is justified by its flexibility in modelling many types of value distributions as indicated by [61]. This function has already been used in CD studies based on SAR images [62,63,64]. The results attained in this area have proven the feasibility of the Weibull distribution. The Maximum Likelihood Estimation (MLE) is applied to estimate the values of the distribution parameters that best fit with the data. This uses the information available in the categories to choose the parameter value (or parameter values) for which such observation is most likely. The expressions of the exponential probability and Weibull functions are Equation (12):

(12) $$f\left(x;\lambda \right)=\lambda e^{-\lambda \cdot x}\;\&\;f(x;\alpha ,\beta )=\frac{\alpha }{\beta^{\alpha }}x^{\alpha -1}e^{-{\left(x/\beta \right)}^{\alpha }},$$

This paper contrasts the results obtained with the previous method based on parametric functions against other results obtained with methods that do not require statistical parameter estimation such as SVM [65], based on the learning theory and considered Heuristic algorithms [66]. Conceptually, the Machine implements the following idea: a set of non-linear vectors map a space of high-dimension characteristics to create a linear decision surface. The special characteristics of this decision surface assure optimal learning of the Machine. In some applications, a pre-classification aimed at transforming the original data into a new feature space with more linearly independent variables correlated with each classifier can be obtained, is suggested by the HSVM algorithm in [67]. Basically and specifically when it comes to image classification, the purpose of SVM is to determine the ideal hyperplane separating two types (binary classifier) using training data. SVM have been extensively described in work by [66] and applied to multispectral image classification [68], as well as to change detection issues [69]. SVM use kernel functions previously defined to transform non-linear (inseparable) decision borders (between types) to linear (separable types) [70]. In this study, the kernels used are Equations (13)–(16):

(13) $$\mathrm{Linear}:\;K(x_{i},x_{j})=x_{i}^{T}x_{j},$$

(14) $$\mathrm{Polynomial}:\;K(x_{i},x_{j})={\left(\gamma x_{i}^{T}x_{j}+r\right)}^{d},\gamma >0,$$

(15) $$\text{Radial Basis Function}:\;K(x_{i},x_{j})=\exp(-\gamma ||(x_{i}x_{j})|{|}^{2},\gamma >0,$$

(16) $$\mathrm{Sigmoid}:\;K(x_{i},x_{j})=\tan\;H(\gamma x_{i}^{T}x_{j}+r),$$

where γ is the width of the kernel function, d is the degree of the polynomial and r is the bias term of the kernel function. The SVM can be applied to CD when the change and no-change are considered a binary classification issue. The usefulness of SVM as changing zone classifiers lies in the possibility of doing a joint analysis of change index images obtained using unsupervised procedures. These images are a part of a multiband image subjected to a supervised binary classification process (change, no-change). The results are CD maps with distinctions between the changing and non-changing areas.

### 2.6. Quality Assessment

Once the different change maps have been obtained with one strategy or another, probabilistic information fusion vs. SVM, the suitability of the results in each of the cases is assessed. This assessment is done using the traditional procedure for selecting check areas and comparing them with the various maps. Of the different quality measurements achieved using this method, this paper not only considers the global accuracy and Kappa coefficient but also the producer and user accuracy.

The producer accuracy (or risk) is related to the error by omission that appears when one pixel that pertains to a certain type is not classified in that type (false negative). The user accuracy (or risk) is related to the error by commission that occurs when a pixel is classified in a category when in all reality it belongs to another (false positive). These measurements represent a significant estimate of the suitability of the results reached although it is an analysis based on pixels and not shapes or objects, which would have a much more real significance. In order to offer the estimate quality of the results greater objectivity and given that work is being done with high-resolution datasets, two metrics were applied for high-resolution images as described in [45,71,72] which involve the use of segmented images.

The first metric considers dividing the image into the background and the foreground and is designated as “Misclassification Error” (ME), which is based on the percentage of cells in the background erroneously assigned to the foreground and conversely, the cells in the foreground erroneously assigned to the background. It is a method that not only assesses the coincidence between categories but also makes it possible to consider the spatial distribution or shape of the objects. For the binary case [72] i.e., change/no_change, the ME is expressed Equation (17) as:

(17) $$\mathrm{ME}=1-\frac{\left|B_{o}\cap B_{k}\right|+\left|F_{o}\cap F_{k}\right|}{\left|B_{o}\right|+\left|F_{o}\right|},$$

where B_o_ and F_o_ are the background and foreground of the original image (ground truth), B_c_ and F_c_ are the values of the background and foreground cells in the classified image, and |.| is the cardinality of the set. The ME varies between 0 and 1 for an erroneously or correctly classified binary image, respectively.

**Figure 6:**
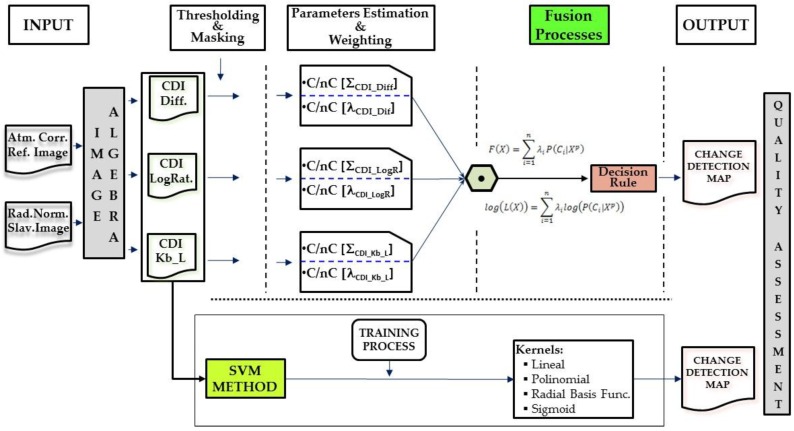
Detailed change detection suggested methodology.

The second, known as “Relative Foreground Area Error” (RAE), is based on a comparison of the properties of the objects such as area and shape (area feature A) obtained from the segmented image with respect to a reference image. The Equation (18) for this measurement is given in [72] as:

(18) $$\mathrm{RAE}=\left\{ \begin{array}{l} \frac{A_{o}-A_{k}}{A_{o}}\;\mathrm{if}\;A_{k}<A_{o}\\ \frac{A_{k}-A_{o}}{A_{o}}\;\mathrm{if}\;A_{k}\geq A_{o} \end{array}\right.,$$

where A_o_ is the area of the reference or check image and A_k_ is the area of the classified or binarized image. For perfect correspondence between the segmented regions, RAE will be 0 while this value will be closer to 1 for a lack of coincidence. Just as the first method uses a set of test pixels distributed evenly throughout the spatial areas of both datasets, the two other quality measurements will be applied to the two selected datasets. The global method followed in this paper is graphically represented in Figure 6.

## 3. Results

The previously explained methodology has been applied to the two SPOT PAN data sets, DS1 and DS2 described in Section 2.1. First, the radiometric normalization of the images of these data sets is achieved, so that the different CDIs are optimally computed; then, the corresponding thresholding and masking operations are executed in order to discriminate change/no-change classes. With these intermediate results, different information metrics are derived, and the statistical characterization of the change and no-change categories is performed. Further on, the probabilistic information fusion process is carried out with the statistical parameters and weighting factors obtained in the preceding stage. In order to assess the goodness of the achieved CD maps by the suggested probabilistic fusion procedure, these outcomes are compared with the results accomplished by the SVM strategy.

### 3.1. Radiometric Correction Process

The radiometric normalization methodology outlined in Figure 5 has been applied to the two sets of data, DS1 and DS2. First, the correct geometric registration of all data sets images has been verified, so that misalignments and other geometric inconsistencies, producing false alarms, are avoided. Once the radiometric process has been carried on, the suitability of the suggested procedure has been assessed by means of the RMSE given in Equation (1), both for relative and absolute radiometric correction strategies.

**Figure 7:**
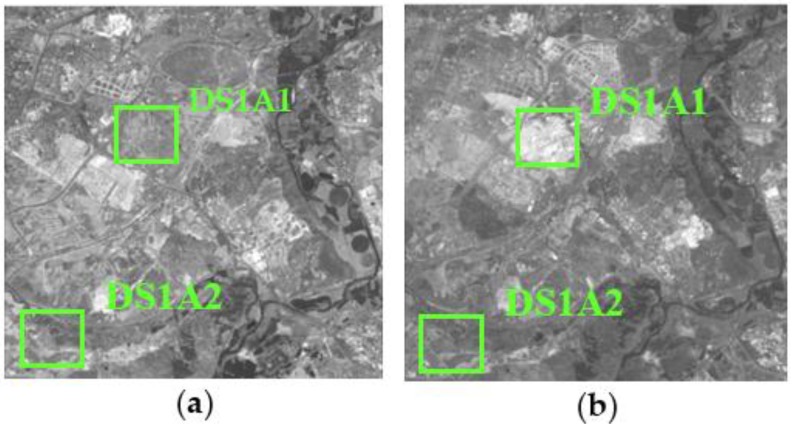
Data set 1 (DS1) with D1SA1 and DS1A2 highlighted subsets, radiometrically normalized images: (**a**) SPOT5 24 July 2005 reference image (master); (**b**) SPOT5 19 July 2008 normalized image (slave).

Figure 7 shows the radiometric corrected DS1 data set, resulting from the proposed methodology. The RMSE for the relative normalization process is 10.75 Digital Numbers (DN), which has decreased to 3.15 DN after the absolute normalization methodology has been completed.

**Figure 8:**
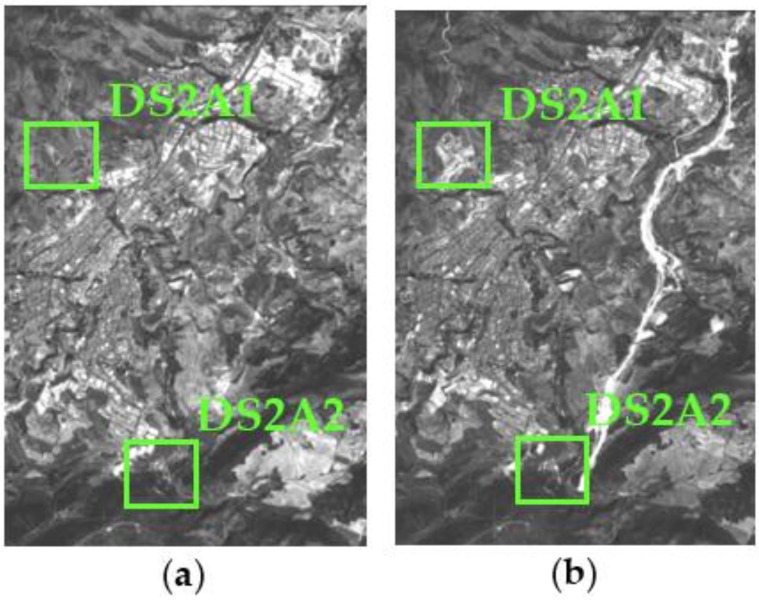
Data set 2 (DS2) with D2SA1 and DS2A2 highlighted subsets, radiometrically normalized images: (**a**) SPOT5 14 August 2005 reference image (master); (**b**) SPOT5 10 August 2008 normalized image (slave).

For the second data set (DS2), the RMSE for the relative radiometric correction process has delivered a RMSE of 3.40 DN. With the suggested absolute radiometric normalization procedure, this value has decreased considerably to 0.87 DN (Figure 8).

Given the size of the processed images, and in order to make a suitable visual analysis in the following sections, two windows or subsets have been selected for this purpose from DS1 and DS2. They are designated by DS1A1, DS1A2, DS2A1 and DS2A2, and are highlighted in Figure 7 and Figure 8.

**Figure 9:**
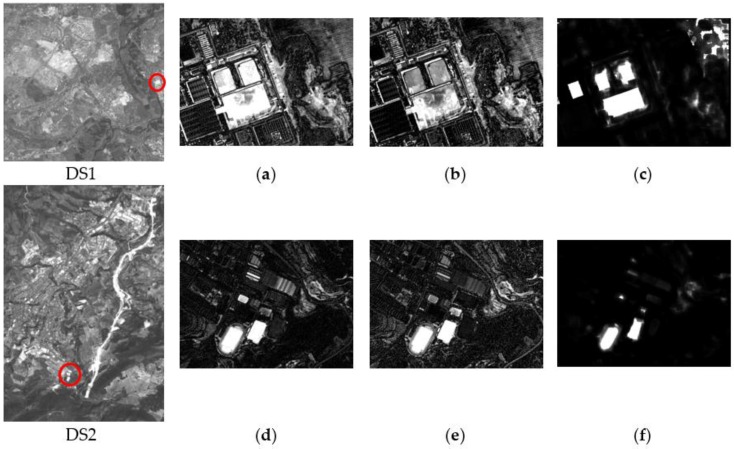
Enlargement for the highlighted areas in the corresponding CDIs in DS1 and DS2: (**a**) DS1-Difference CDI; (**b**) DS1-Log_Ratio CDI; (**c**) DS1-Kb_Leibler CDI; (**d**) DS2-Difference CDI; (**e**) DS2-Log_Ratio CDI; (**f**) DS2-Kb_Leibler CDI.

The radiometric corrected DS1 and DS2 images have been used for deriving the three CDIs specified in Section 2.3, i.e., image differencing, Log_Ratio and Kb_Leibler distance. As it was mentioned in that section, the idea behind processing distinct CDIs is to take advantage of the additional information contained in these products for improving the outcome of a CD process. This complementary is confirmed by observing the differences between the three CDIs considered in this study. For this purpose, two small zones (Figure 9) have also been selected from DS1 and DS2, so that the mentioned differences can be appreciated.

### 3.2. CDI Thresholding

In this work, image thresholding has been implemented automatically based on the criteria stated in Section 2.2 and Section 2.3, by which a particular thresholding algorithm is selected based on the data range of a specific image, in this case a CDI. In both data sets, the optimal binary maps have been obtained by applying the Otsu method for the Difference and Log_Ratio CDIs, while the Renyi method appeared to be more suitable for the Kb_Leibler CDI. These derived binary maps or images play an important role in the subsequent processes of the probabilistic information fusion procedure, as they allow to automatically discriminating the change/no-change range of values in each CDI. Then, with the corresponding derived values for these categories, it is possible to compute their information content, as well as estimating the best probability functions fitting these distributions. These two processes are described in the next two sections.

### 3.3. Information Content Evaluation, Weights Assignement

Thus, the estimated intervals of values for the two considered categories in each CDI, have been used for evaluating their information content. This has been performed with several information metrics as specified in Section 2.4. The corresponding expressions for these metrics are given in Equations (5)–(7). First, the Informational or Shannon Entropy (H(Glob)) has been computed for each CDI in DS1 and DS2. Then, this metric has been applied to each CDI category separately. This metric is designated here by H(C_i_) with C_i_ = C or C_i_ = nC. The second metric applied in this work has been the Conditional Entropy, which is designated by H(X/C_i_). The last considered metric is the Specific Information or SI(C_i_) of Equation (7). The results of the information content evaluation for DS1 and DS2 are reported in Table 1 and Table 2. For DS1, different information content values have been reached for each CDI and their corresponding categories, as observed in Table 1. It is worth to note that the lower the entropy, the greater the informational content. However, for Specific Information (SI) the higher the value, the higher the contribution of the variable or category.

In this data set, the Difference and Kb_Leibler CDIs exhibit a higher information content (H(Glob)) compared to the Log_Ratio CDI. However, this scenario changes when this metric is evaluated for each category individually (H(C) or H(nC)). Again, this circumstance also changes when considering Conditional Entropies (H(X/C) or H(X/nC)). For Specific Information, the achieved values differ once more in comparison with other metrics values. However, it can be noted that the value reached for the change category in the Kb_Leibler CDI (SI(C) = −2.3), clearly indicates the lowest contribution compared to the two other CDIs. This lower contribution is also observed for H(C) and H(X/C). In these cases, the no-change category exhibits a similar behavior.

Analogous considerations can be extended to the results of this analysis in DS2 (Table 2). However, some differences can also be noted. The Shannon entropy assigns the highest information content to the Kb_Leibler CDI (H(Glob) = 1.67). For the same CDI, when considering the involved categories individually, this metric also provides de maximum amount of contribution (CDI, H(C) = 0.45 and H(nC) = 1.21) compared to contribution of the difference and Log_Ratio CDIs. However, these values differ again for the Conditional Entropy and Specific Information metrics. In both cases, the information content derived from these two metrics, maintains the same trend already observed for DS1.

Once the values for the different metrics have been derived, the following step consists in assigning weights to each CDI as mentioned in Section 2. The corresponding results or weights are reported in Table 3 and Table 4.

For DS1, the weights agree with the contribution expressed by each individual metric (Table 3). It is important to note that these new values rank each CDI and categories by their contribution. Thus the lower entropy values lead to highest weights. Conversely, a lower Specific Information implies a lower weight, which is the case for the change category in the Kb_Leibler CDI (λ_SI(C)_ = 0.04).

Similarly, the same trends and behaviors are observed for DS2 (Table 4). At this stage of the study, it is important to outline, that all these weights have been assigned analytically based on three types of information metrics.

Further on in this work, weights λ_H(C)_ and λ_H(nC)_ are not taken into account for the fusion process as they exhibit in most cases similar values to λ_H(X/C)_ and λ_H(X/nC)_. This also enables to reduce the number of study cases. In order to assess the impact on the change maps, the weights are introduced in Equations (9) and (11) in combination with the statistical parameters corresponding to probabilities functions that best describe the categories of each CDI.

### 3.4. Adjustment of Parameters and Statistical Analysis

From the change/no-change values defined by the thresholded CDIs, the most likely parameters for the distribution functions specified in Section 2.5 are estimated. For this reason, the probability functions and associated parameters describing the most conveniently those change/no-change values in each CDI, are verified, i.e., Gauss, Exponential and Weibull.

**Figure 10:**
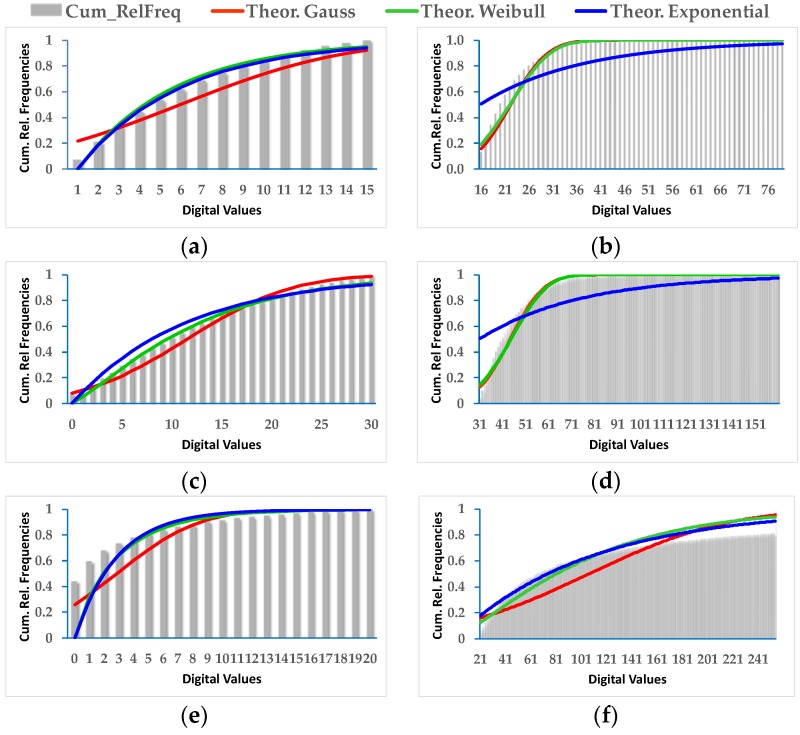
DS1, adjusted Gauss, exponential and Weibull probability distributions: (**a**) No_Change CDI Difference; (**b**) Change CDI Difference; (**c**) No_Change CDI Log_Ratio; (**d**) Change CDI Log_Ratio; (**e**) No_Change CDI Kb_Leibler; (**f**) Change CDI Kb_Leibler.

For DS1, depending on the CDI and the considered category, all three probabilistic functions exhibit acceptable adjustments (Figure 10). Table 5 shows the results achieved for the estimated parameters of the Gauss and Weibull density probability functions, i.e., mean (µ) and deviation (σ), as well as Shape (α) and Scale (β) values. The RMSE of the adjustment or fit can also be observed. This value enables to identify the most appropriate probability function for the two categories of a given CDI. This RMSE is derived using the corresponding theoretical Gaussian or Weibull distribution functions. For this purpose the estimated parameters have been considered, in addition with the cumulative relative frequency histograms of the change/no-change category values of each CDI. According to the results presented in Table 5, from the six considered situations, four of them are best approximated by the Weibull function, in contrast to the Gaussian probability function which only fits properly in two cases, thus confirming the fact that the Gaussian probability function is not always the most convenient.

In order to strengthen these observations, for each CDI and related change/no-change category values in DS1, all probability functions, theoretical and experimental (cumulative histograms), are represented in Figure 10, where the exponential distribution function is also considered. The results for the exponential function are not presented in Table 5, since under these circumstances, either it behaves similarly to the Weibull or is clearly not feasible, as it can be appreciated in Figure 10.

For the second data set (DS2), very similar results to DS1 are achieved. However, as it is observed in Table 6, there are generally some few differences between the RMSEs that might confirm one or the other function for approximating the change/no-change values. Nevertheless, for further analysis, the parameters of the best fitted probability functions have always been considered.

In spite of these findings, although certain functions are more appropriated for a particular CDI change/no-change category, it must be clarify that these categories are not necessarily of that probability function type.

### 3.5. Implementation of the Probabilistic Information Fusion

The implementation of the probabilistic information fusion models Equations (9) and (11) has taken into account both, the Gauss and Weibull probabilities functions and their associated parameters reported in Section 3.4, as well as the reliability factors or weights derived in Section 3.3. Regarding the latter, and in order to draw conclusions about the information metrics, the following four types of weights are considered for performing the fusion processes: no weights (λ_Nw_), global weights (λ_Glob_), conditional weights (λ_H(X/C)_, λ_H(X/nC)_) and Specific Information weights (λ_SI(C),_ λ_SI(nC)_). The results are different CD maps obtained by combining different probability functions and weights.

**Figure 11:**
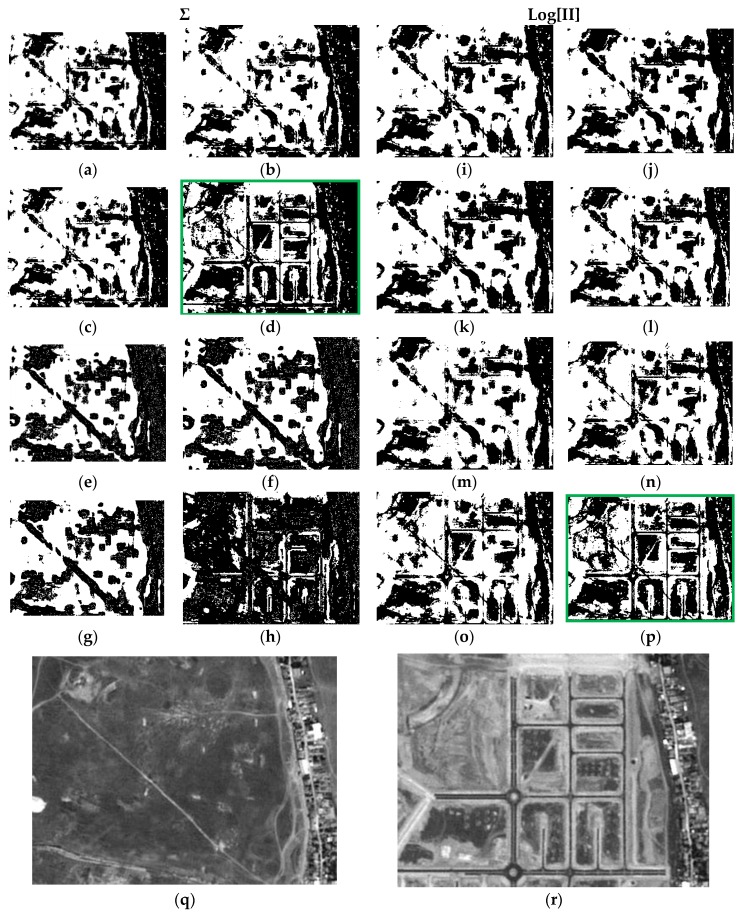
Probabilistic Information Fusion Results in DS1A1: (**a**) c1; (**b**) c2; (**c**) c3; (**d**) c4; (**e**) c5; (**f**) c6; (**g**) c7; (**h**) c8; (**i**) c9; (**j**) c10; (**k**) c11; (**l**) c12; (**m**) c13; (**n**) c14; (**o**) c15; (**p**) c16; (**q**) DS1A1 2005 SPOT-PAN image; (**r**) DS1A1 2008 SPOT-PAN image.

Each result is denoted by a case “c#”, and a total of 16 cases have been derived for each data set, e.g., case c1 is the result of applying Equation (9) or LOP model, with Gaussian probability functions (G) and respective parameters (Table 5 and Table 6) for the two categories (C and nC) of each CDI. Moreover, in case c1 weights have not been applied (λ_Nw_ = 1). All the considered cases are summarized in Table 7. The first 16 results corresponding to each concerned case of Table 7 are presented in Figure 11.

For the sake of simplicity and clarity, only subset DS1A1 (Figure 7) for these outcomes is displayed in this Figure. It can be noted that cases c4 and c16 (green boxes) show more consistent changes compared to those observed in the SPOT radiometrically corrected image subsets of Figure 11q,r. These two results are basically identical. It must be noted that case c4 has been generated with Gaussian functions and associated parameters with Specific Information weights (λ_SI(C)_, λ_SI(nC)_). Although, the result shown in c16 has also been obtained applying the same type of weights, the best fitting probability functions specified in Table 5 were instead applied. In all remaining CD maps, the change category (white pixels) is overly represented, except for c8, where a loss of information for this category is observed (Figure 11h).

**Figure 12:**
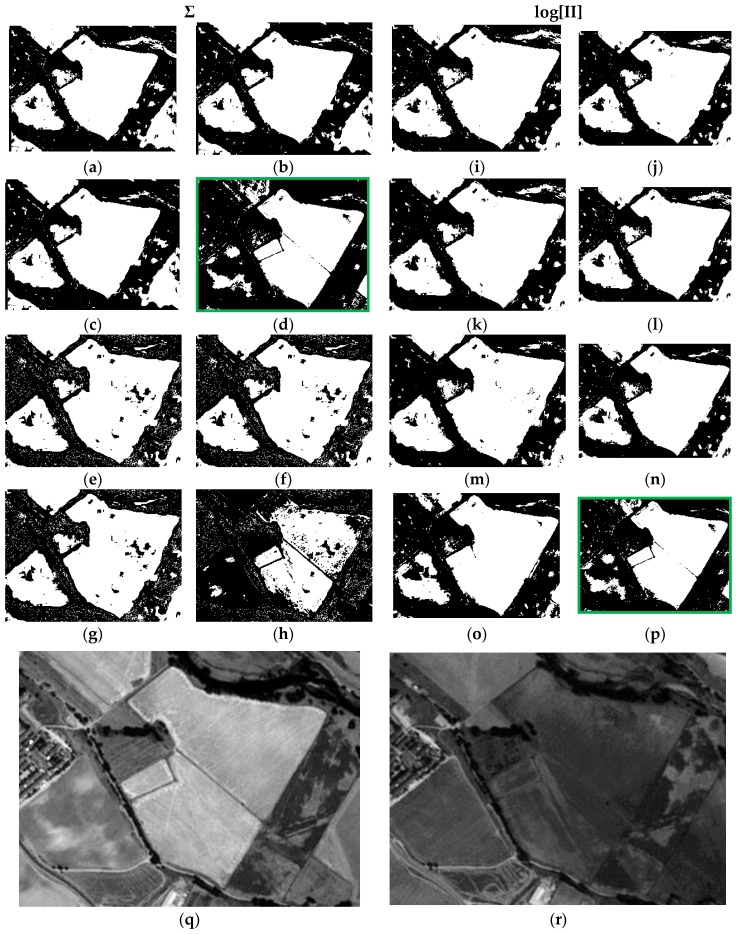
Probabilistic Information Fusion Results in DS1A2: (**a**) c1; (**b**) c2; (**c**) c3; (**d**) c4; (**e**) c5; (**f**) c6; (**g**) c7; (**h**) c8; (**i**) c9; (**j**) c10; (**k**) c11; (**l**) c12; (**m**) c13; (**n**) c14; (**o**) c15; (**p**) c16; (**q**) DS1A2 2005 SPOT-PAN image; (**r**) DS1A2 2008 SPOT-PAN image.

Precisely the same remarks can be extended to the second test area of the first test dataset (DS1A2), which results are displayed in Figure 12.

**Figure 13:**
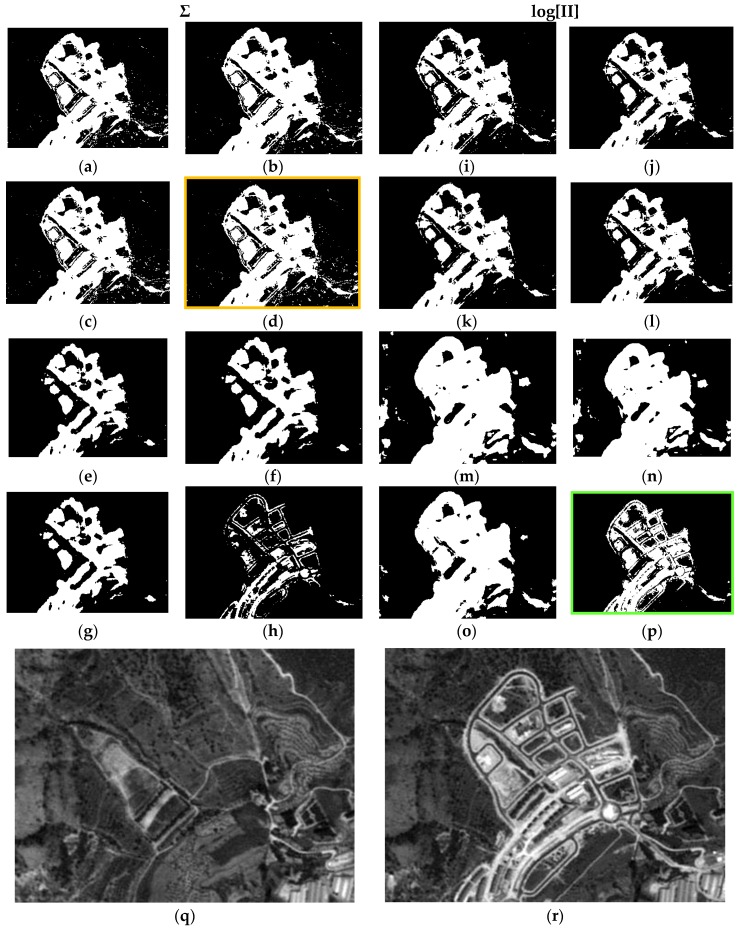
Probabilistic Information Fusion Results in DS2A1: (**a**) c1; (**b**) c2; (**c**) c3; (**d**) c4; (**e**) c5; (**f**) c6; (**g**) c7; (**h**) c8; (**i**) c9; (**j**) c10; (**k**) c11; (**l**) c12; (**m**) c13; (**n**) c14; (**o**) c15; (**p**) c16; (**q**) DS2A1 2005 SPOT-PAN image; (**r**) DS2A1 2008 SPOT-PAN image.

For the second data set (DS2) and first test area (DS2A1), similar behaviors to those observed in either test areas or subsets of DS1 are also verified. Only the fourth case (c4, orange box) exhibits some differences, i.e., an excess of change pixels is observed, when compared to the respective cases in DS1 and with the two SPOT reference images (Figure 13q,r), and with case c16, as well. However, this latter, is in complete accordance with its homologue cases (c16) in Figure 11p and Figure 12p. For case c8 (Figure 13h), the same deficiencies are also observed, i.e., complete loss of information.

**Figure 14:**
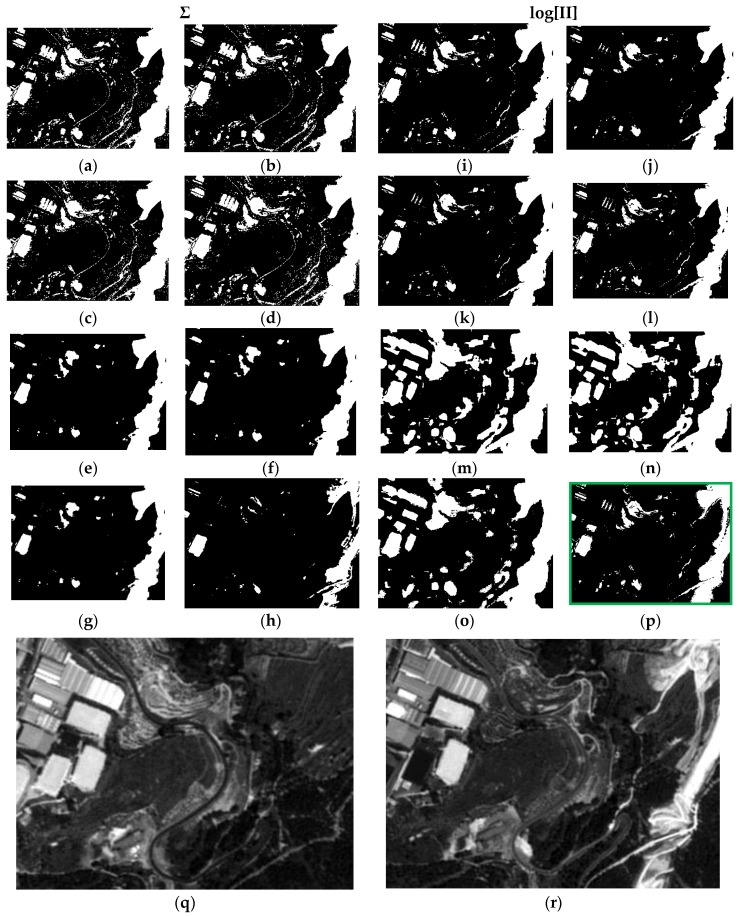
Probabilistic Information Fusion Results in DS2A2: (**a**) c1; (**b**) c2; (**c**) c3; (**d**) c4; (**e**) c5; (**f**) c6; (**g**) c7; (**h**) c8; (**i**) c9; (**j**) c10; (**k**) c11; (**l**) c12; (**m**) c13; (**n**) c14; (**o**) c15; (**p**) c16; (**q**) DS2A2 2005 SPOT-PAN image; (**r**) D2S2A2 2008 SPOT-PAN image.

Finally, all the remarks formulated previously are also extended to the results of the last test area (DS2A2) (Figure 14). Again, the result shown in c16 (Figure 14), which has been computed applying the weights from the Specific Information metric with the best adjusted parameters and corresponding pdf’s, as well as with the LOGP procedure, has delivered the best graphic results. The remaining cases are also similar to those observed in Figure 13, and particularly c4 (Figure 14h). Therefore, the best configuration of weights, probability functions and procedures, is that corresponding to case c16.

### 3.6. SVM Fusion

**Figure 15:**
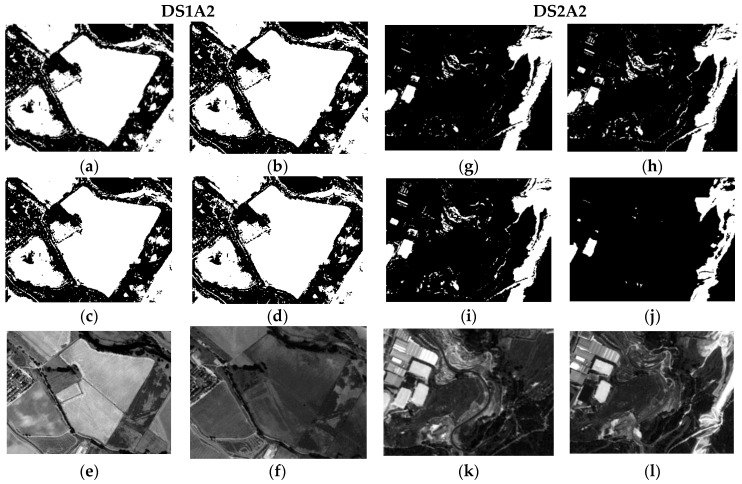
SVM classification results in DS1A2 and DS2A2: (**a**) DS1A2 linear; (**b**) DS1A2 polynomial; (**c**) DS1A2 RBF; (**d**) DS1A2 sigmoid; (**e**) DS1A2 2005 SPOT-PAN image; (**f**) DS1A2 2008 SPOT-PAN image; (**g**) DS2A2 linear; (**h**) DS2A2 Polynomial; (**i**) DS2A2 RBF; (**j**) DS2A2 sigmoid; (**k**) DS2A2 2005 SPOT-PAN image; (**l**) DS2A2 2008 SPOT-PAN image.

Each group of CDIs in the first (DS1) and second (DS2) datasets constituted as multiband images, have been classified by means of the four Support Vector Machine (SVM) Kernels specified in Section 2.5. The training of this classifier has been supervised by an analyst who has selected groups of change/no-change pixels throughout the two data sets. For DS1, a total number of 103,178 training pixels have been selected, and 9412 for DS2. The common parameters established for all selected kernels are: a penalty parameter, a pyramid level and the classification probability threshold value. The first parameter is set in all cases at its maximum value, 1000, which requires all training pixels to converge to a certain class. The pyramid level is set to a value of zero for all kernels, so the image is processed with its spatial resolution, which also produces a decrease in processing speed. Finally, a threshold parameter is required for avoiding unclassified pixels. Figure 15 shows an example of the SVM classification results for DS1 and DS2, which can also be compared, for the same test areas or subsets, with the outcomes of the probabilistic fusion procedures. The different SVM kernels deliver quite similar results. Only the sigmoid kernel in the second data set (DS2A2) exhibit some differences compared to the other three. With regard to the probabilistic fusion procedure and the respective test areas (DS1A2 and DS2A2), these results are inferior compared to the achieved cases c16.

### 3.7. Results of Quality Assessment

For assessing the different results, two sets of check areas have created respectively for DS1 and DS2, which have been randomly distributed throughout both datasets. For this purpose, 12,360 pixels have been selected in DS1 and 1612 pixels for DS2.

First, the results for the assessment of the SVM classification results using these check areas for are shown in Table 8 and Table 9, respectively. As it is observed all SVM classification kernels have produced good results in terms of overall accuracies and Kappa coefficient in both data sets. Only the accuracy values for the SVM sigmoid kernel are lower.

Regarding the probabilistic fusion procedures, the 16 resulted CD maps in both data sets have also been assessed. Table 10 shows the accuracy values for DS1. For 12 of the 16 cases, global accuracies near to 94% are reached, i.e., a high percentage of success. Only four change maps, resulting from the LOP fusion process exhibit values below 90%, but still above 80%, which can be considered sufficiently high.

The same trend is also observed for the Kappa Coefficient values, although the higher value do not exceeds 0.9 and never attains 1, which is its maximum value. Thus, this metric appears to be a more realistic measure as it better accounts for the number of errors represented by the producer and user accuracies, whose values are also available on Table 10.

Although, this metric (Kappa Coef.) is also able to identify the most suitable results (c4 and c16), it does not differentiate them properly from other graphical inadequate cases, e.g., c5 or c6 among others. Likewise, an additional important issue detected in Table 10, is the low overall accuracy attained for c16, compared to other cases whose graphical appearance clearly showed certain deficiencies. This remark also holds for the Kappa metric. So, for this particular case both accuracies values were unexpected, and the question is whether these quality metrics and methodology are appropriate for such type of data.

The above comments may be extended to the results shown in Table 11 for the second data set (DS2). In this situation, the global accuracies reach practically 100% and the Kappa coefficient is close to 1.

Thereby, in spite of these acceptable accuracy results which show a correct estimation of the performance of a particular method, it is considered that these accuracy metrics are not entirely realistic, since they do not properly take into account the precise form of the objects contained in high resolution images. Thus, the pixel based accuracy estimation procedure is not considered to be the most suitable for these types of images. Instead, it is suggested to apply methods founded on the so called Object Based Image Analysis (OBIA), such as the Misclassification Error (ME) and the Relative Area Error (RAE) explained in Section 2.6 and given in Equations (17) and (18), respectively.

**Figure 16:**
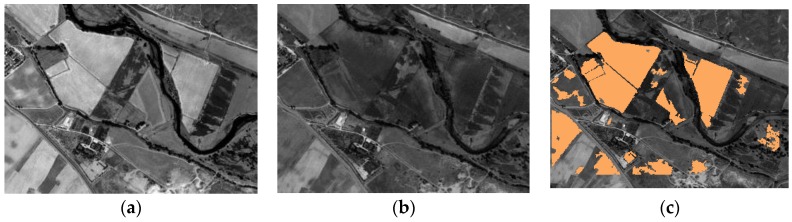
DS1 subset example for object based analysis: (**a**) 2005 SPOT-PAN image subset; (**b**) 2008 SPOT-PAN image subset; (**c**) Test objects.

This process has been performed on the first data set (DS1) for the probabilistic fusion procedure, where shapes or objects such as those shown in Figure 16c have been delineated. Further on, these forms are rasterized, so that they can be converted into check areas and thus properly combined with the respective CD maps.

**Figure 17:**
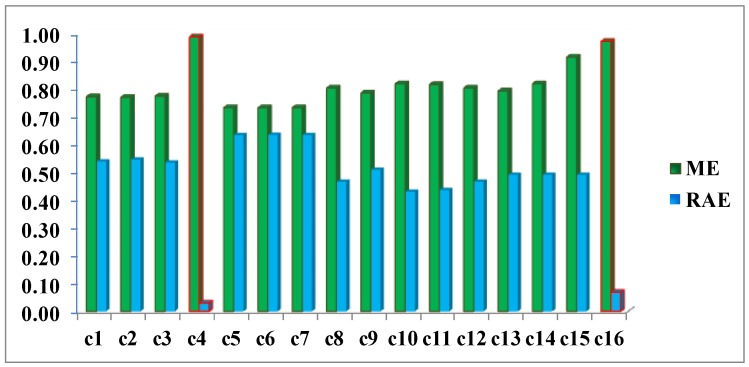
Missclosure (ME) and Relative Area (RAE) Errors for DS1 change maps.

Once, these preliminary operations have been performed, it is possible to count the cardinality of the two involved categories, required for applying the two quality metrics ME and RAE. The accomplished results are reported in Figure 17. Both measures have ranges of values between 0 and 1. For ME a value close to 1 expresses the highest quality, whereas the opposite occurs for the RAE metric.

This example clearly highlights how metrics ME and RAE point out cases 4 and 16 as the best CD maps and verify quantitatively the findings resulting from the visual analysis of the images displayed in Figure 12. These errors (red bars in Figure 17) are 0.99 and 0.97, respectively, for the ME metric, and 0.03 and 0.07 for RAE. This confirms that these two quality control metrics are more objective measures, compared to the traditional regular and uniformly distributed test areas, as they inherently take into account the shape of the objects. Although, the two best cases or results (c4 and c16) have been measured correctly with the ME metric, they are better discriminated from the remaining deficient cases by the RAE, therefore this latter seems to be better quality measure.

This procedure has not been performed for the SVM methods, as false positives and negatives are perfectly localized, in addition to the CD map differences observed between the SVM results in Figure 15 and the parametric results for cases c4 and c16 in Figure 12 and Figure 14.

## 4. Discussion

The radiometric normalization method applied differs from others methodologies in the use of a threshold image calculated automatically which is used to define the pseudo invariant features (PIF). These features are zonal features in this paper. The RMSE obtained for the two datasets, 3.15 and 0.87, respectively, confirm the validity of the method proposed. This clearly shows that it is not necessary to apply overly sophisticated atmospheric corrections for images acquired by SPOT HRG sensors operating in Panchromatic mode and that the generic method DOS (Dark Object Subtraction) is sufficient. Thus, the Change indices calculated with these transformed datasets can be expected to more reliably and accurately represent the changes and no-changes that occur in a land scene. This is basically what occurred with the three considered CDIs as can be seen in Figure 9. Although they tend to represent common changes, it can be very clearly observed in each one that they also present complementary information. Hence, one of the aims of this study consists of analytically assessing the quantity of information or the contribution of each CDI to a CD process. In this case, the work is based on the use of probabilistic information fusion, which requires the assignment of weights based on the quantity of information contained in the CDIs.

The evaluation of the quantity of information was done by testing three informational metrics. Although all three are based on Shannon entropy, each one represents different informational quantities. Initially, Specific Information (SI) proves to be the most adequate of the different metrics applied in comparison to Informational Entropy and Conditional Entropy. After analyzing the values of the CDI Kb_Leibler in the two datasets, the weight 0.04 assigned for the change category in both sets was initially considered correct and will be proven further below via the experiments completed. The weights 0.56 and 0.28 were also quite adequate for the no-change category in DS1 and DS2, as will be outlined below.

As concerns the CD processes, a method based on the multi-sensor probabilistic information fusion theory was first analyzed. The choice of this method is due to the fact that it seems quite appropriate for integrating different CDIs with different contributions represented by the weights corresponding to each CDI. Although they were calculated based on a pair of images acquired by a single sensor, each CDI is considered to be the resulting information acquired by independent sensors from a probabilistic perspective. This is justified in that each CDI is calculated based on a mathematical expression with absolutely different terms and parameters.

As a prior phase to applying the fusion method, the change/no-change categories must be parametrized with some type of probability function. Of the three probability functions initially analyzed, only the Gauss and Weibull functions were taken into consideration given that the exponential function behaves much like the Weibull function or simply does not fit. It has been proven that the Gauss function is not always the most appropriate although it may be applicable in some cases. The Weibull function is better for most situations.

As a result of the above, it is possible to evaluate the two probabilistic information fusion models. For the LOP method, acceptable results are generally produced as per the contingency tables (Table 10 and Table 11). For case c4 (Figure 11, Figure 12, Figure 13 and Figure 14), it results that many of the false alarms disappear by applying the SI, leading to a higher quality change map. This is also described to a lesser extent by the values of the two quality metrics ME and RAE represented for this same case (case 4) in Figure 17, 0.97 and 0.03, respectively, which indicates better performance of all the experiments.

Contrary to what this work set out to prove and always using the LOP fusion procedure, the observation from the experiments done was that the change maps generated based on other probabilistic distribution functions—not necessarily Gaussian ones—did not produce the desired results. This demonstrates that, although in most cases the Weibull adapts betters to the change/no-change categories for the summative probabilistic method, the results in general are not convincing as can be seen in Figure 11, Figure 12, Figure 13 and Figure 14, even though the global accuracy values (Table 10 and Table 11) show high values for this metric. Nonetheless, the RAE metric distinguishes case c4 as the most accurate, correctly discriminating it with respect to the other cases in this family.

These considerations can be equally extended to the LOGP fusion procedure. For this method, most of the experiments completed can be considered unsatisfactory. The second best result c16, very close to c4, with a RAE = 0.07 (vs. 0.03 for c4) and an ME = 0.97 (vs. 0.99 for c4) was reached by applying the logarithmic method and the weights deriving from the Specific Information (SI) metric. Therefore and in view of these values, working with distribution functions that best describe the different categories is considered an absolutely correct supposition even though it is not a sufficient condition for reaching acceptable results and they were obtained by applying the weights calculated with the SI metric when it is decisive for both models in achieving good results. Therefore, this also proves that the SI along with the choice of probability functions, which are not necessarily Gaussian, is also a correct and rigorous solution versus the alternative of considering the change/no-change categories with Gaussian parameters, when the weights are also properly chosen.

Likewise, the fact that more than one CDI was used made it possible to enhance the final result with the complementary information each one of them contributes. Although the CDI Kb_Leibler tends to generalize the forms of the change categories, its participation in this work in the fusion process made it possible to locate no-change areas which in and of themselves would have been detected by means of the other two CDIs, whereas this CDI seems rather deficient for the change category and was correctly identified by the SI and the corresponding weights.

Finally and in general, the method based on probabilistic information fusion may be considered capable of producing better results than the different SVM Kernels evaluated, as long as the parameters and weights are properly identified.

## 5. Conclusions

An information fusion method used to detect changes in images obtained from panchromatic sensors must be based on three essential pillars. Firstly, a test of a modified absolute radiometric normalization method, which enables minimizing the radiometric differences between two images acquired by the same sensor at different times. This test obviously requires that the images of the data set are appropriately co-registered. Secondly, the different information metrics that make it possible to calculate weights to weigh the CDIs that intervene in the fusion must duly quantify each one’s contribution and their categories. The third pillar must be the evaluation of a probability function, so that the statistical parameters are ideally adjusted to the change/no-change categories in order to be used in the selected probabilistic information fusion models. Weighted logarithmic probabilistic fusion (LOGP) offers a completely reliable alternative to non-parametric procedures such as Support Vector Machine (SVM).

Although CD Indices from a single source or sensor have been traditionally used to detect changes, others obtained from physically different sensors may be used as would be the case of radar antennae or thermal sensors in order to establish a multisource fusion method. To this end, the probabilistic method seems to be better adapted or more flexible for fusing with other information sources that enable a determination of the type of change.

## Figures and Tables

**Table 1:** DS1, CDI Information (metrics) Values.

CDI	H(Glob)	H(C)	H(nC)	H(X/C)	H(X/nC)	SI(C)	SI(nC)
Difference	4.3	1.0	3.3	4.2	3.7	0.1	0.6
Log_Ratio	5.4	1.3	4.1	5.1	4.7	0.3	0.7
Kb_Leibler	4.3	1.7	2.6	6.6	3.0	−2.3	1.7

**Table 2:** DS2, CDI Information (metric) Values.

CDI	H(Glob)	H(C)	H(nC)	H(X/C)	H(X/nC)	SI(C)	SI(nC)
Difference	2.91	0.58	2.33	4.02	2.41	−1.11	0.50
Log_Ratio	3.83	0.87	2.96	4.48	3.17	−1.57	0.66
Kb_Leibler	1.67	0.45	1.21	6.41	1.21	−4.74	0.46

**Table 3:** DS1, Weights (λ) corresponding to CDI Information (metric) Measures.

CDI	λ_Glob_	λ_H(C)_	λ_H(nC)_	λ_H(X/C)_	λ_H(X/nC)_	λ_SI(C)_	λ_SI(nC)_
Difference	0.36	0.42	0.32	0.40	0.33	0.46	0.23
Log_Ratio	0.28	0.33	0.26	0.33	0.24	0.50	0.21
Kb_Leibler	0.36	0.25	0.41	0.27	0.43	0.04	0.56
Σ	1	1	1	1	1	1	1

**Table 4:** DS2, Weights (λ) corresponding to CDI Information (metric) Measures.

CDI	λ_Glob_	λ_H(C)_	λ_H(nC)_	λ_H(X/C)_	λ_H(X/nC)_	λ_SI(C)_	λ_SI(nC)_
Difference	0.28	0.34	0.27	0.40	0.27	0.51	0.30
Log_Ratio	0.22	0.22	0.21	0.35	0.20	0.45	0.41
Kb_Leibler	0.50	0.44	0.52	0.25	0.53	0.04	0.28
Σ	1	1	1	1	1	1	1

**Table 5:** DS1, PDF Parameters.

CDI	Category	Gauss Par. (G)	Weibull Par. (Wb)	RMSE	Best Fit
Difference	No-change	µ = 4.95	α = 1	7.10 × 10^−5^ (G)	Weibull
		σ = 6.37	β = 4.67	2.43 × 10^−5^ (Wb)	
	Change	µ = 21.15	α = 3.53	1.80 × 10^−4^ (G)	Weibull
		σ = 6.15	β = 23.26	1.66 × 10^−4^ (Wb)	
Log_Ratio	No-change	µ = 11.53	α = 1.2	5.53 × 10^−5^ (G)	Weibull
		σ = 8.27	β = 12.92	3.01 × 10^−5^ (Wb)	
	Change	µ = 44.18	α = 4.05	1.83 × 10^−4^ (G)	Gauss
		σ = 11.63	β = 48.71	6.14 × 10^−3^ (Wb)	
Kb_Leibler	No-change	µ = 2.85	α = 0.9	1.01 × 10^−4^ (G)	Gauss
		σ = 4.41	β = 2.9	1.31 × 10^−4^ (Wb)	
	Change	µ = 107.22	α = 1.23	6.95 × 10^−4^ (G)	Weibull
		σ = 87.34	β = 109.44	4.91 × 10^−4^ (Wb)	

**Table 6:** DS2, PDF Parameters.

CDI	Category	Gauss Par. (G)	Weibull Par. (Wb)	RMSE	Best Fit
Difference	No-change	µ = 1.61	α = 0.31	1.72 × 10^−4^ (G)	Weibull
		σ = 2.46	β = 0.32	1.55 × 10^−4^ (Wb)	
	Change	µ = 13.57	α = 2.43	3.00 × 10^−4^ (G)	Weibull
		σ = 5.92	β = 15.1	2.20 × 10^−4^ (Wb)	
Log_Ratio	No-change	µ = 5.76	α = 1.3	4.70 × 10^−5^ (G)	Weibull
		σ = 3.67	β = 6.2	4.56 × 10^−5^ (Wb)	
	Change	µ = 21.21	α = 2.48	0.96 (G)	Weibull
		σ = 9.39	β = 24	0.96 (Wb)	
Kb_Leibler	No-change	µ = 0.65	α = 0.14	2.77 × 10^−4^ (G)	Gauss
		σ = 2.09	β = 0.0004	3.47 × 10^−4^ (Wb)	
	Change	µ = 63.02	α = 1.14	2.73 (G)	Weibull
		σ = 66.43	β = 66.75	2.73 (Wb)	

**Table 7:** Summary of the different considered cases.

Case Nr.	Fus. Mod. Σ = (9) log(II) = (11)	Probabilistic Functions (G = Gaussian, W = Weibull)												Weights
		Data Set 1 (DS1)						Data Set 2 (DS2)						
		CDI Diff.		CDI LogR.		CDI Kb_L		CDI Diff.		CDI LogR.		CDI Kb_L		
		C	nC	C	nC	C	nC	C	nC	C	nC	C	nC	
c1	Σ	G	G	G	G	G	G	G	G	G	G	G	G	λ_Nw_
c2	Σ	G	G	G	G	G	G	G	G	G	G	G	G	λ_Glob_
c3	Σ	G	G	G	G	G	G	G	G	G	G	G	G	λ_H(X/C)_/λ_H(X/nC)_
c4	Σ	G	G	G	G	G	G	G	G	G	G	G	G	λ_SI(C)_/λ_SI(nC)_
c5	Σ	W	W	G	W	W	G	W	W	W	W	W	G	λ_Nw_
c6	Σ	W	W	G	W	W	G	W	W	W	W	W	G	λ_Glob_
c7	Σ	W	W	G	W	W	G	W	W	W	W	W	G	λ_H(X/C)_/λ_H(X/nC)_
c8	Σ	W	W	G	W	W	G	W	W	W	W	W	G	λ_SI(C)_/λ_SI(nC)_
c9	Log(II)	G	G	G	G	G	G	G	G	G	G	G	G	λ_Nw_
c10	Log(II)	G	G	G	G	G	G	G	G	G	G	G	G	λ_Glob_
c11	Log(II)	G	G	G	G	G	G	G	G	G	G	G	G	λ_H(X/C)_/λ_H(X/nC)_
c12	Log(II)	G	G	G	G	G	G	G	G	G	G	G	G	λ_SI(C)_/λ_SI(nC)_
c13	Log(II)	W	W	G	W	W	G	W	W	W	W	W	G	λ_Nw_
c14	Log(II)	W	W	G	W	W	G	W	W	W	W	W	G	λ_Glob_
c15	Log(II)	W	W	G	W	W	G	W	W	W	W	W	G	λ_H(X/C)_/λ_H(X/nC)_
c16	Log(II)	W	W	G	W	W	G	W	W	W	W	W	G	λ_SI(C)_/λ_SI(nC)_

**Table 8:** DS1. Accuracy analysis of the SVM Fusion process.

SVM Kernel ZONE 1	Producer Acc. (%)		User Acc. (%)		G. Acc (%)/Kappa Coef.
	C	NC	C	NC	
SVM linear	98.74	84.73	90.97	97.73	93.26/0.85
SVM polynomial	98.72	84.96	91.09	97.72	93.34/0.86
SVM Rad. Bas. Funct. (RBF)	98.71	85.29	91.27	97.70	93.46/0.86
SVM sigmoid	98.75	84.63	90.91	97.75	93.23/0.85

**Table 9:** DS2. Accuracy analysis of the SVM Fusion process.

SVM Kernel ZONE 2	Producer Acc. (%)		User Acc. (%)		G. Acc (%)/Kappa Coef.
	C	NC	C	NC	
SVM linear	86.55	98.40	97.63	90.60	93.29/0.86
SVM polynomial	87.25	95.11	93.12	90.76	91.72/0.83
SVM Rad. Bas. Funct. (RBF)	86.97	95.00	92.96	90.57	91.54/0.83
SVM sigmoid	68.07	100.0	100.0	80.48	86.21/0.71

**Table 10:** DS1. Accuracy analysis of the Probabilistic Information Fusion process.

Fusion Process DS1	Producer Acc. (%)		User Acc. (%)		G. Acc (%)/Kappa Coef.
	C	NC	C	NC	
c1	96.51	89.33	93.37	94.26	93.70/0.87
c2	96.52	89.30	93.36	94.28	93.70/0.87
c3	96.49	89.28	93.34	94.24	93.67/0.87
**c4**	98.14	88.19	92.82	96.82	94.83/0.89
c5	96.77	90.73	94.21	94.75	94.41/0.88
c6	95.60	92.26	95.06	93.09	94.30/0.88
c7	95.75	91.89	94.84	93.28	94.24/0.88
c8	96.52	90.86	94.26	94.37	94.30/0.88
c9	87.95	79.52	86.99	80.91	84.65/0.68
c10	88.09	79.48	86.99	81.09	87.72/0.68
c11	88.20	79.52	87.02	81.23	84.81/0.68
c12	80.23	83.29	88.20	73.01	81.42/0.62
c13	93.45	89.80	93.45	89.80	92.02/0.83
c14	96.36	91.35	94.55	94.16	94.40/0.88
c15	96.33	91.95	94.91	94.15	94.62/0.89
**c16**	94.82	91.89	94.79	91.93	93.67/0.87

**Table 11:** DS2, Accuracy analysis of the Probabilistic Information Fusion process.

Fusion Process DS2	Producer Acc. (%)		User Acc. (%)		G. Acc (%)/Kappa Coef.
	C	NC	C	NC	
c1	100	99.56	99.43	100	99.75/0.99
c2	100	99.56	99.43	100	99.75/0.99
c3	100	99.56	99.43	100	99.75/0.99
c4	100	99.56	99.43	100	99.75/0.99
c5	95.58	99.56	99.41	96.69	97.83/0.96
c6	95.44	99.56	99.41	96.59	97.77/0.95
c7	95.87	99.56	99.41	96.90	97.95/0.96
c8	90.88	100	100	93.43	96.03/0.92
c9	99.57	99.56	99.43	99.67	99.57/0.99
c10	97.29	99.56	99.42	97.95	98.57/0.97
c11	98.86	99.56	99.43	99.12	99.26/0.98
c12	99.57	99.89	99.86	99.67	99.75/0.99
c13	97.44	99.56	99.42	98.05	98.63/0.97
c14	97.29	99.56	99.42	97.95	98.57/0.97
c15	97.29	99.56	99.42	97.95	98.57/0.97
**c16**	94.82	91.89	94.79	91.93	99.32/0.99
